# Supplementary material for: Cryo-EM structure of human eIF5A-DHS complex reveals the molecular basis of hypusination-associated neurodegenerative disorders
Source: Nat Commun. 2023 Mar 27;14:1698. doi: 10.1038/s41467-023-37305-2 (PMC10042821; doi:10.1038/s41467-023-37305-2)
Supplement: Supplementary file 1 — Supplementary Information [file 41467_2023_37305_MOESM1_ESM.pdf]

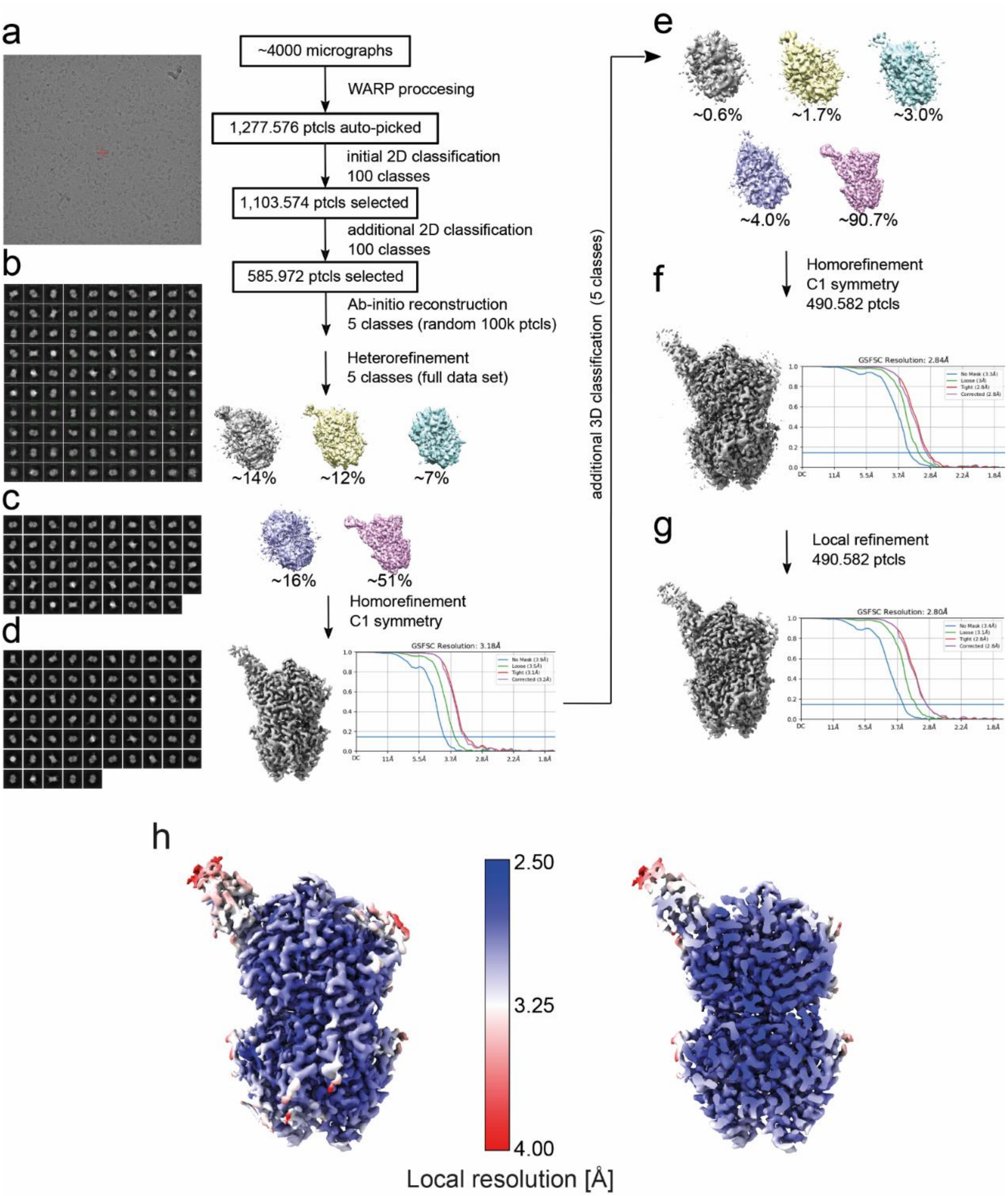

Supplementary Fig. 1. **Computational steps for Cryo-EM reconstruction: A schematic representation.** (**a**) representative micrograph, (**b**) initial reference-free 2D classes, (**c**) first selected 2D classes, (**d**) final selected 2D classes, (**e**) final result of the 3D classification with the percentage of the particles, (**f**) homogenous refinement result of the selected 3D volume (left) with FSC curve (right), (**g**) final result of the reconstruction after Local refinement (left) together with FSC curve (right); the resolution is reported at the gold standard FSC value of 0.143; reconstruction was performed using cryoSPARC v3.3.1. (**h**) Local resolution coloring of the reconstructed volume is presented in the surface representation (left) and cross-section (right) with the local resolution range scale (middle). CryoEM-reconstructed volumes are contoured at the level of RMSD = 3.5.

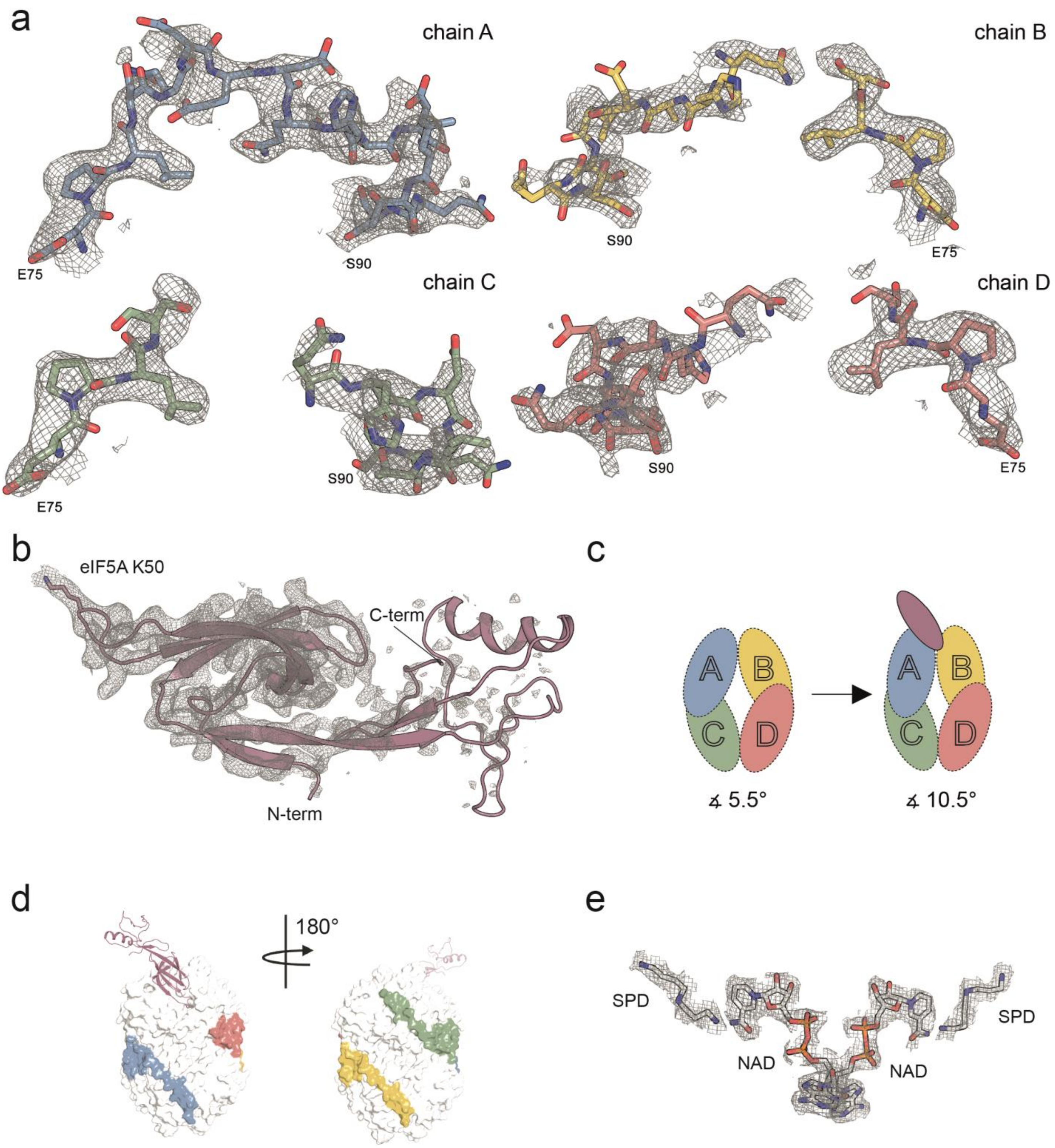

Supplementary Fig. 2. **Additional aspects of microscopic reconstruction.** (**a**) EM map (mesh) quality in a DHS region is known from crystallographic analyses to be very flexible for all chains of a tetramer. (**b**) Quality of the EM map (mesh) for eIF5A bound to DHS; clearly, the N-terminal domain interacting with DHS is better described. (**c**) Schematic representation of the quaternary changes in DHS after binding to eIF5A. The angle between monomers A and B increases by approx. 5 degrees (as calculated by ChimeraX) whilst the relative orientation of monomers C and D (opposite from the binding site) remained virtually unaltered. (**d**) The *ball-and-chain* motifs wind around the DHS and are visible for all chains except chain D, in which the N-terminal helix and a large part of the linker would collide with the bound eIF5A and are therefore dissociated from the surface and flexible to an extent beyond traceability. (**e**) Quality of the electron microscopy map around the small molecule ligands.

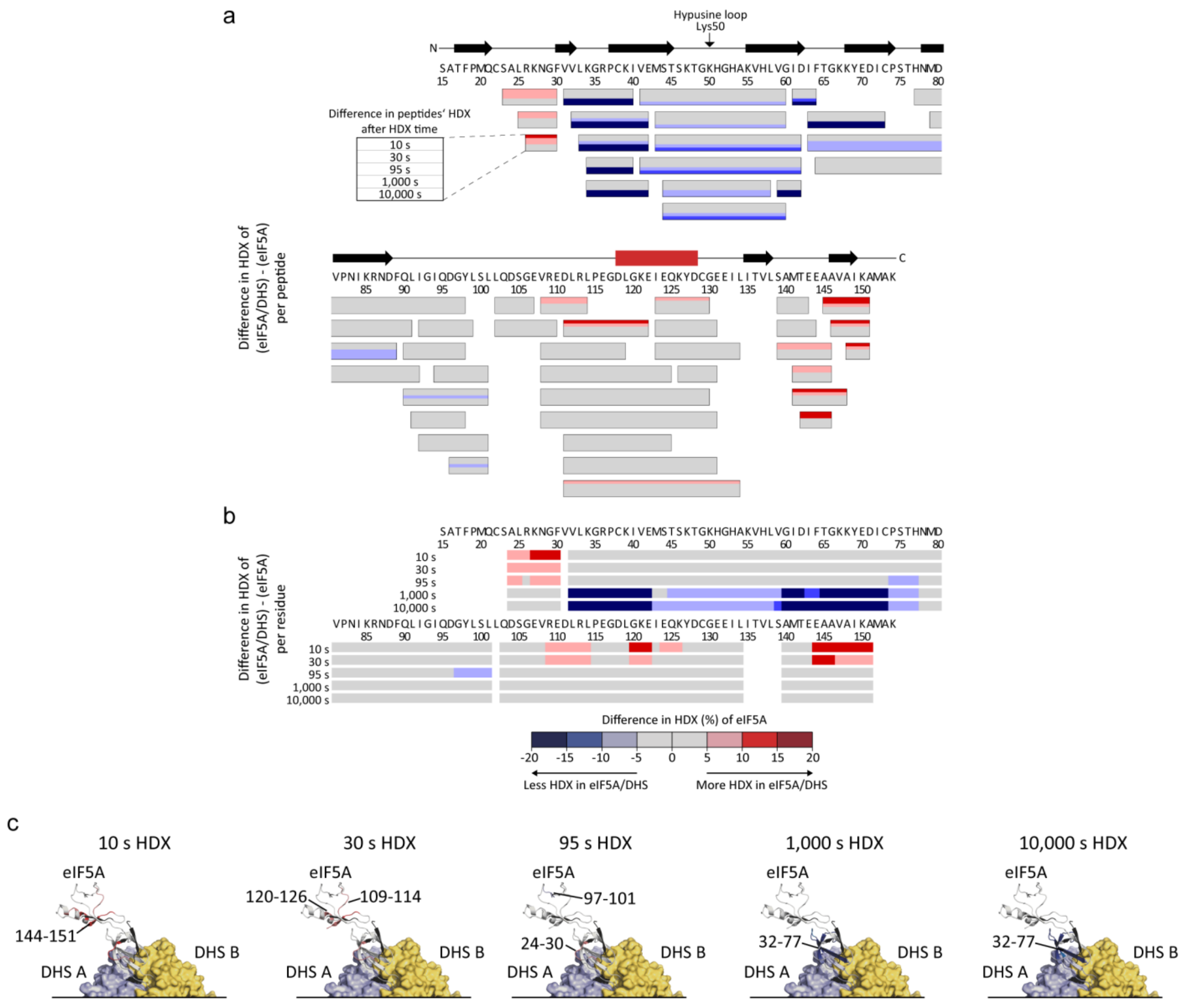

Supplementary Fig. 3. Conformational changes of eIF5A evidenced by HDX-MS. (a, b) The difference in HDX between the eIF5A/DHSK329A complex and individual eIF5A is displayed **(a)** per peptide, and **(b)** per residue of eIF5A. In **(a)** each black box represents an eIF5A peptide identified in HDX-MS. The cartoon depicts the secondary structure (red box, α-helix; black arrow, β-strand) of eIF5A as per the structure of the eIF5A/DHSK329A complex, and depicts the position of Lys50 contained in the hypusine loop. **(c)** The difference in HDX of eIF5A between the eIF5A/DHS^{K329A} complex and individual eIF5A at each timepoint of HDX is projected onto the structural model of the complex. Two DHS^{K329A} monomers are shown in surface representation and colored in blue/yellow, eIF5A is shown in cartoon representation and colored per difference in HDX per residue as in (b). Residues not covered by peptides are colored in black.

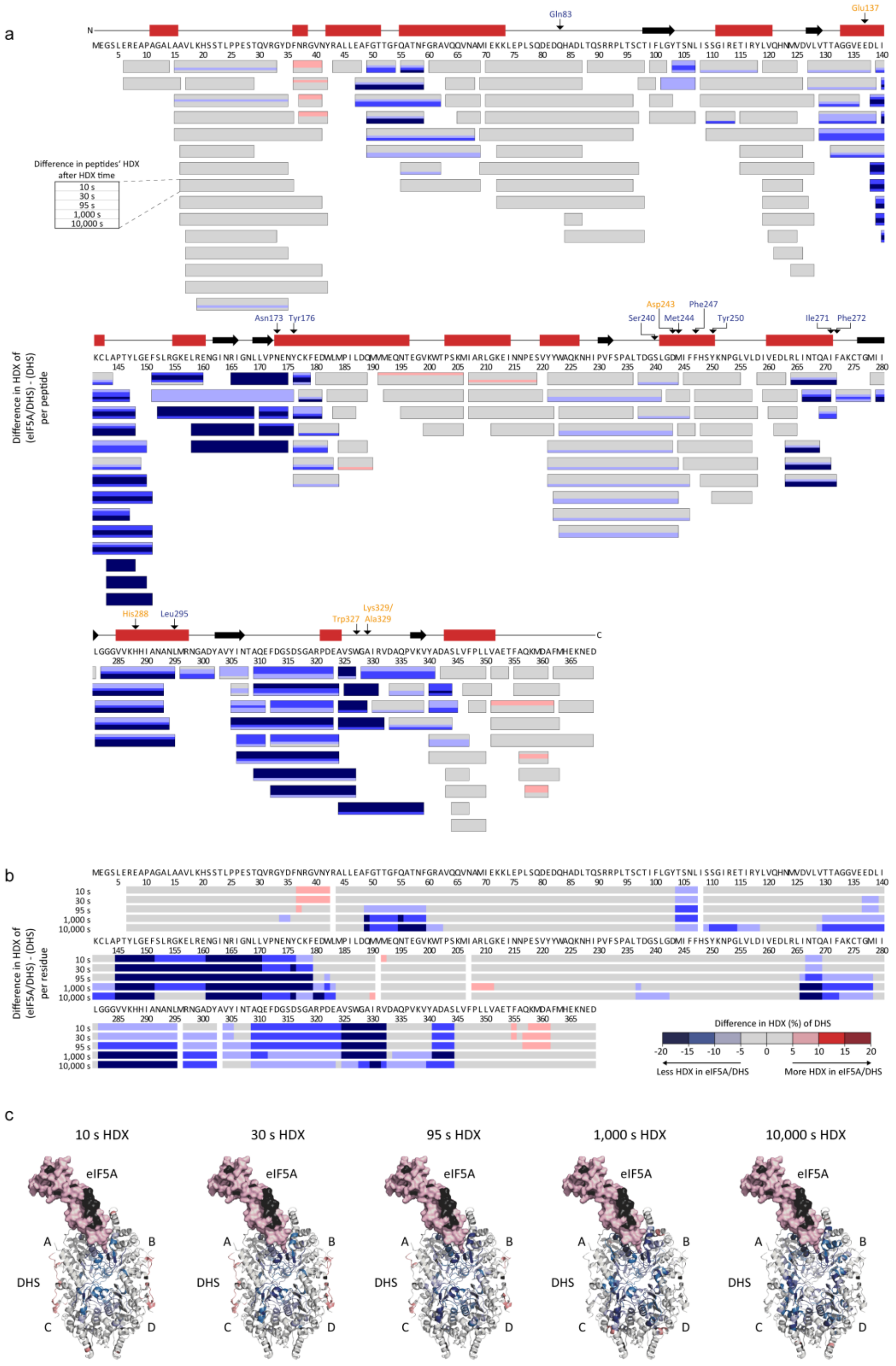

Supplementary Fig. 4. Conformational changes of DHS evidenced by HDX-MS. (a, b) The difference in HDX between the eIF5A/DHS^{K329A} complex and individual DHS^{K329A} is displayed **(a)** per peptide, and **(b)** per residue of DHS^{K329A}. In **(a)** each black box represents a DHS^{K329A} peptide identified in HDX-MS. The cartoon depicts the secondary structure (red box, α-helix; black arrow, β-strand) of DHS^{K329A} as per the structure of the eIF5A/DHS^{K329A} complex, and depicts the positions of active site residues (orange) and residues participating in the coordination of eIF5A (blue). **(c)** The difference in HDX of DHS^{K329A} between the eIF5A/DHS^{K329A} complex and individual DHS^{K329A} at each timepoint of HDX is projected onto the structural model of the complex. DHS^{K329A} is shown in cartoon representation and colored per difference in HDX per residue as in (**b**), eIF5A is shown in surface representation and colored in pink. Residues not covered by peptides are colored in black.

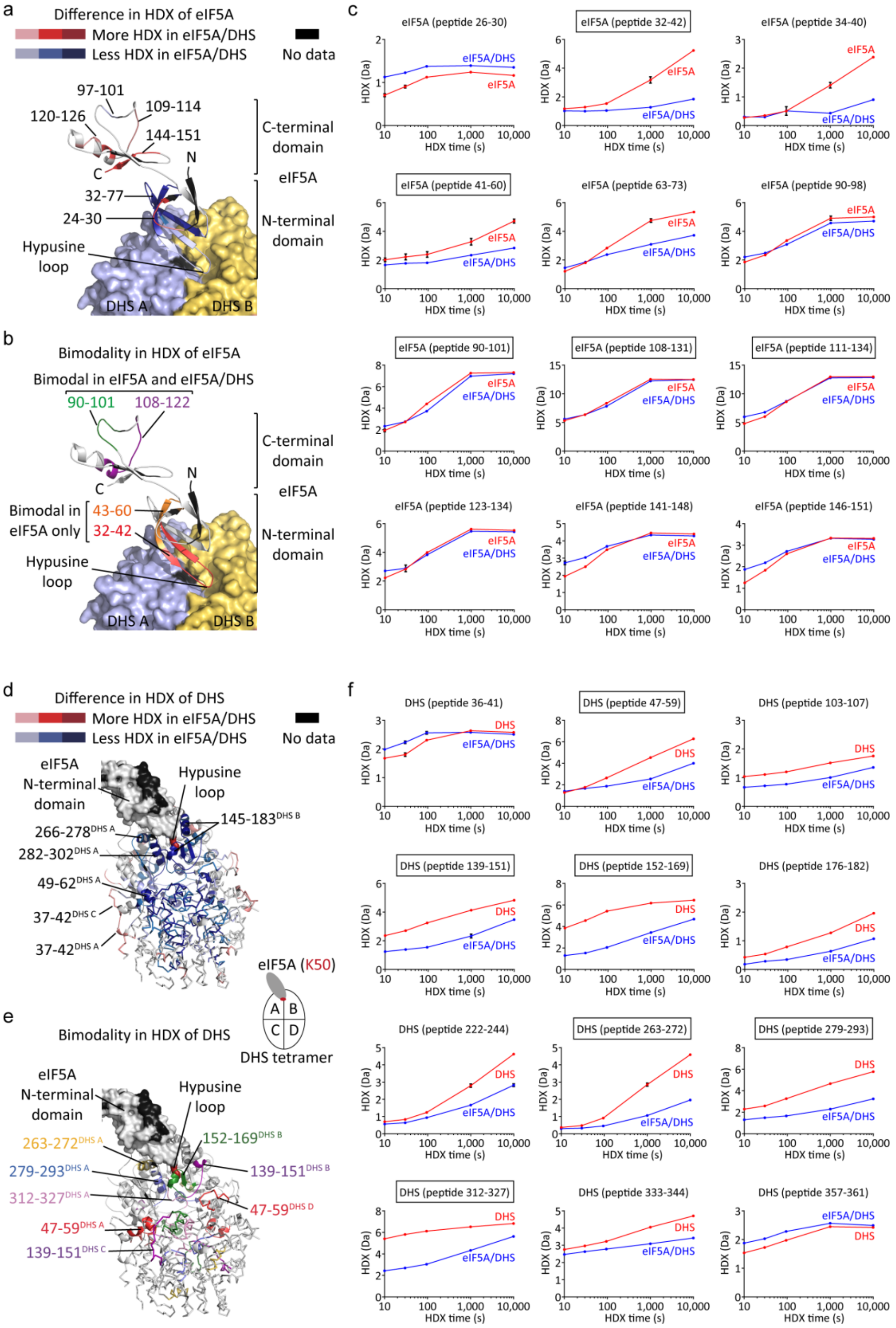

Supplementary Fig. 5. Conformational flexibility of DHS and eIF5A. (**a, d**) Differences in HDX of eIF5A (**a**) and DHS^{K329A} (**d**) between their complex and the individual proteins (compare to Ext. Data Fig. 3&4) are displayed on the structure of the eIF5A/DHS^{K329A} complex. The highest difference observed at any timepoint of HDX was projected (**b, e**) Areas of eIF5A (**b**) and DHS^{K329A} (**e**) that apparently exhibited bimodality of HDX are displayed on the structure of the eIF5A/DHS^{K329A} complex. Mass spectra for bimodal peptides are given in Supplementary Fig. 6 & 7 for eIF5A and DHS^{K329A}, respectively. (**c, f**) HDX of representative peptides of eIF5A (c) and DHS^{K329A} (**f**) of the proteins in isolation (red curves) or in the eIF5A/DHS^{K329A} complex (blue curves). Residue numbers are given in brackets. Black boxes indicate peptides for which a bimodal distribution of HDX was apparent (compare to Supplementary Fig. 6-7). Data represent mean ± SD of n=3 technical replicates.

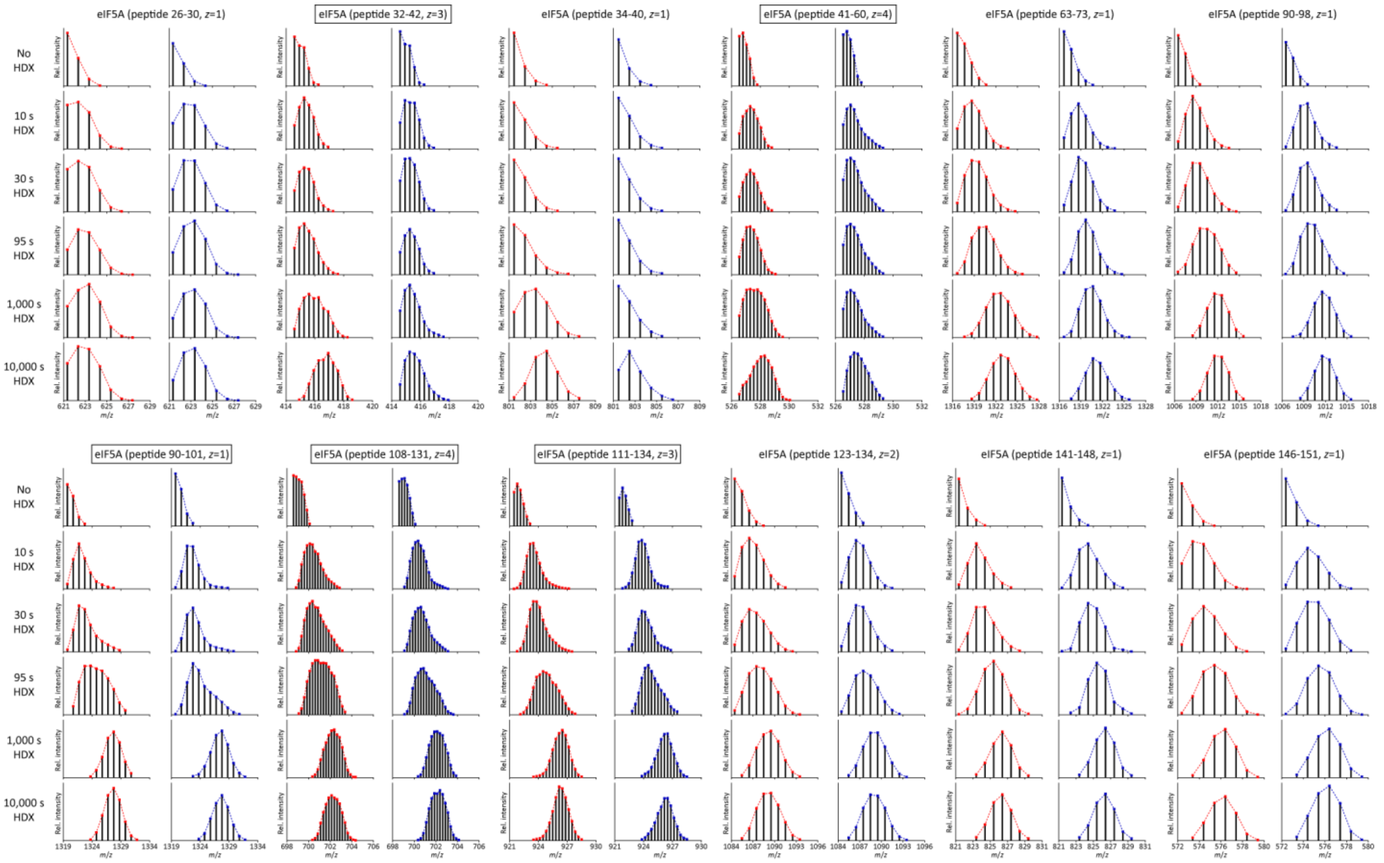

Supplementary Fig. 6. Bimodality in HDX of eIF5A. Mass spectra of representative eIF5A peptides (shown as peptide ion sticks) in individual eIF5A (red) or the eIF5A/DHSK329A complex (blue). Brackets denote the covered residues and charge state for each peptide. Black boxes indicate peptides for which EX1 or mixed EX1/EX2* kinetics of HDX were apparent.

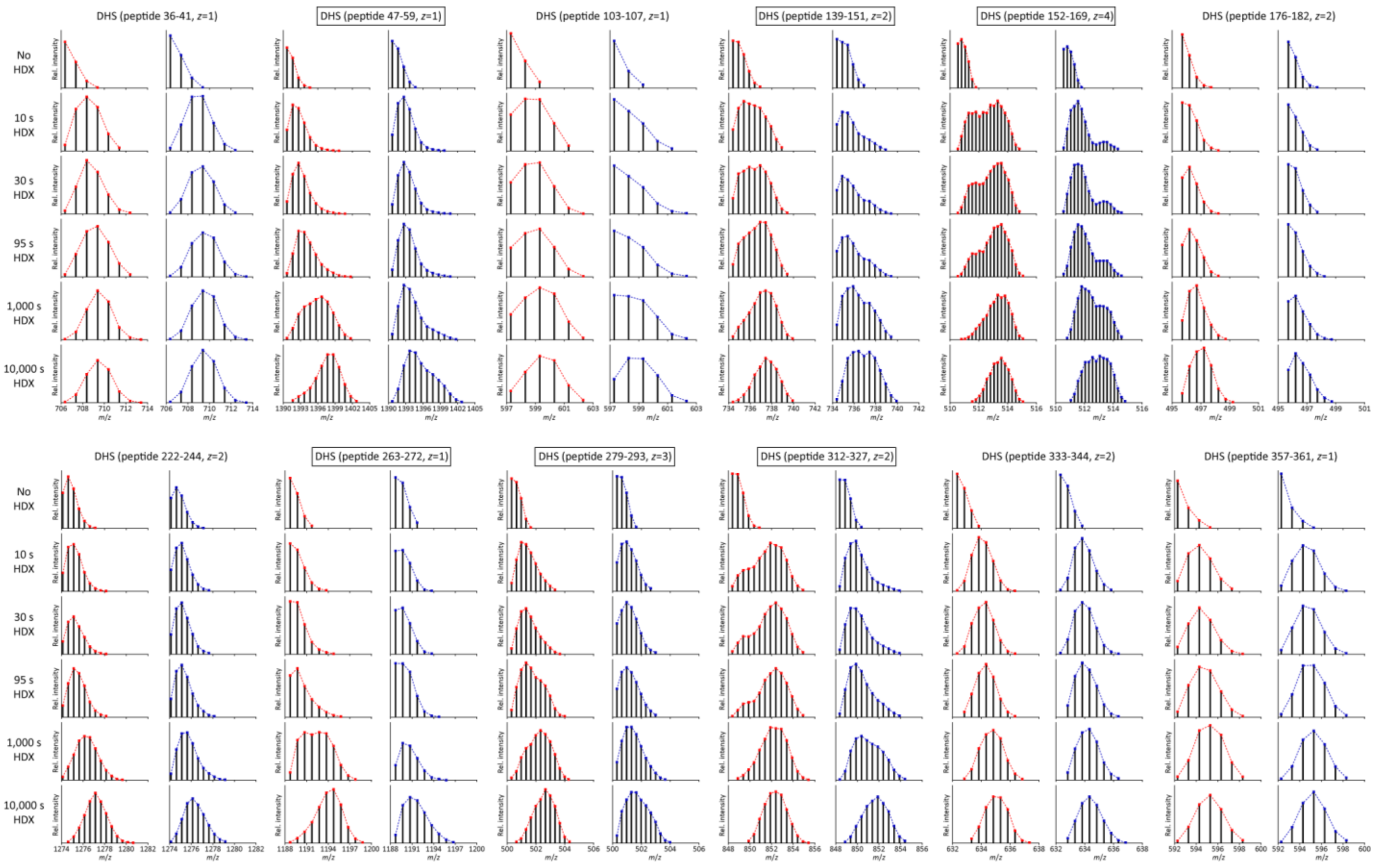

Supplementary Fig. 7. Bimodality[1] in HDX of DHS^{K329A}. Mass spectra of representative DHS^{K329A} peptides (shown as peptide ion sticks) in individual DHS^{K329A} (red) or the eIF5A/DHS^{K329A} complex (blue). Brackets denote the covered residues and charge state for each peptide. Black boxes indicate peptides for which EX1 or mixed EX1/EX2 kinetics of HDX were apparent.

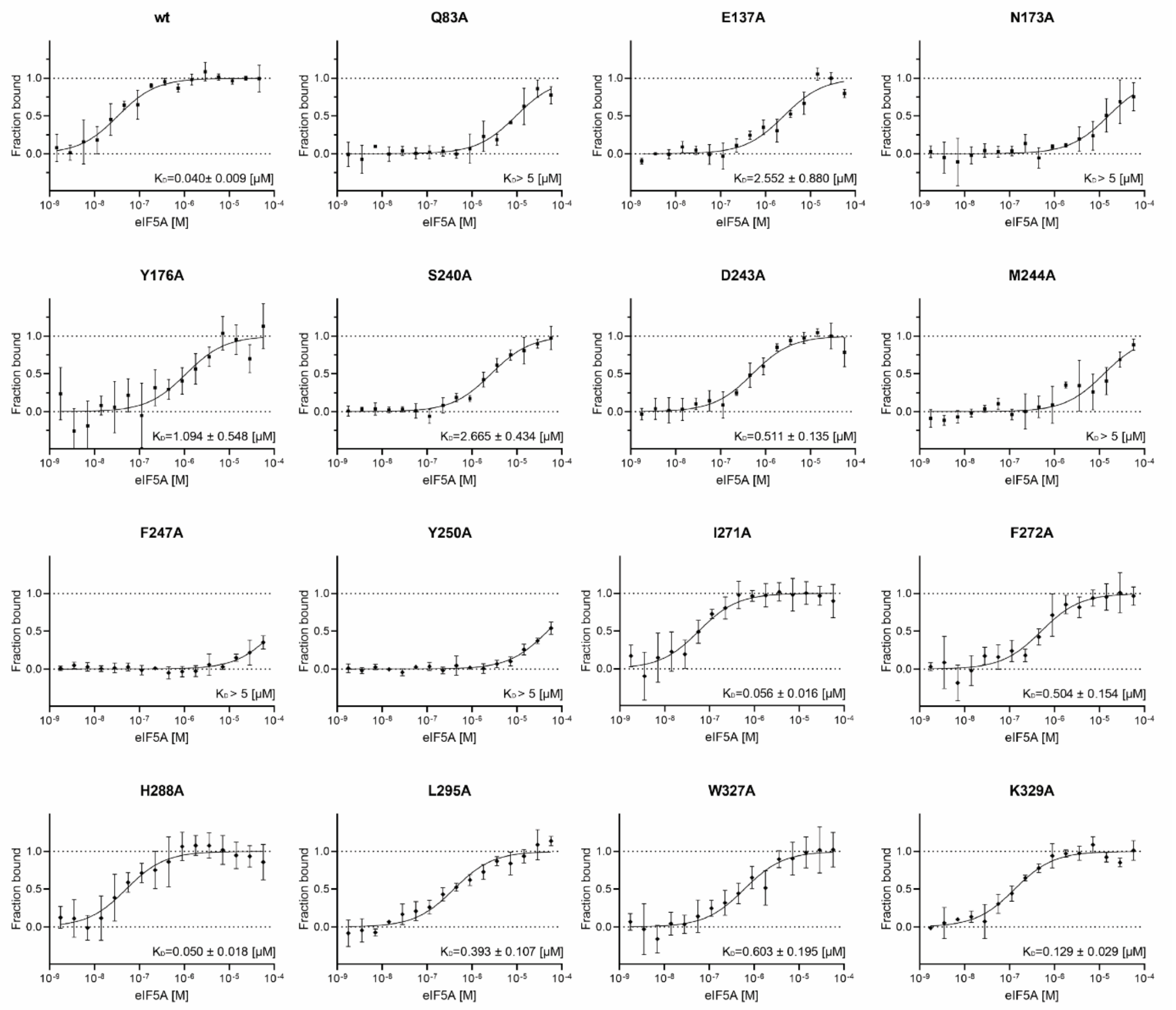

Supplementary Fig. 8. Raw data for MST experiments. Data represent the mean ± SD of n=3 individual experiments. Source data are provided in Source Data File.

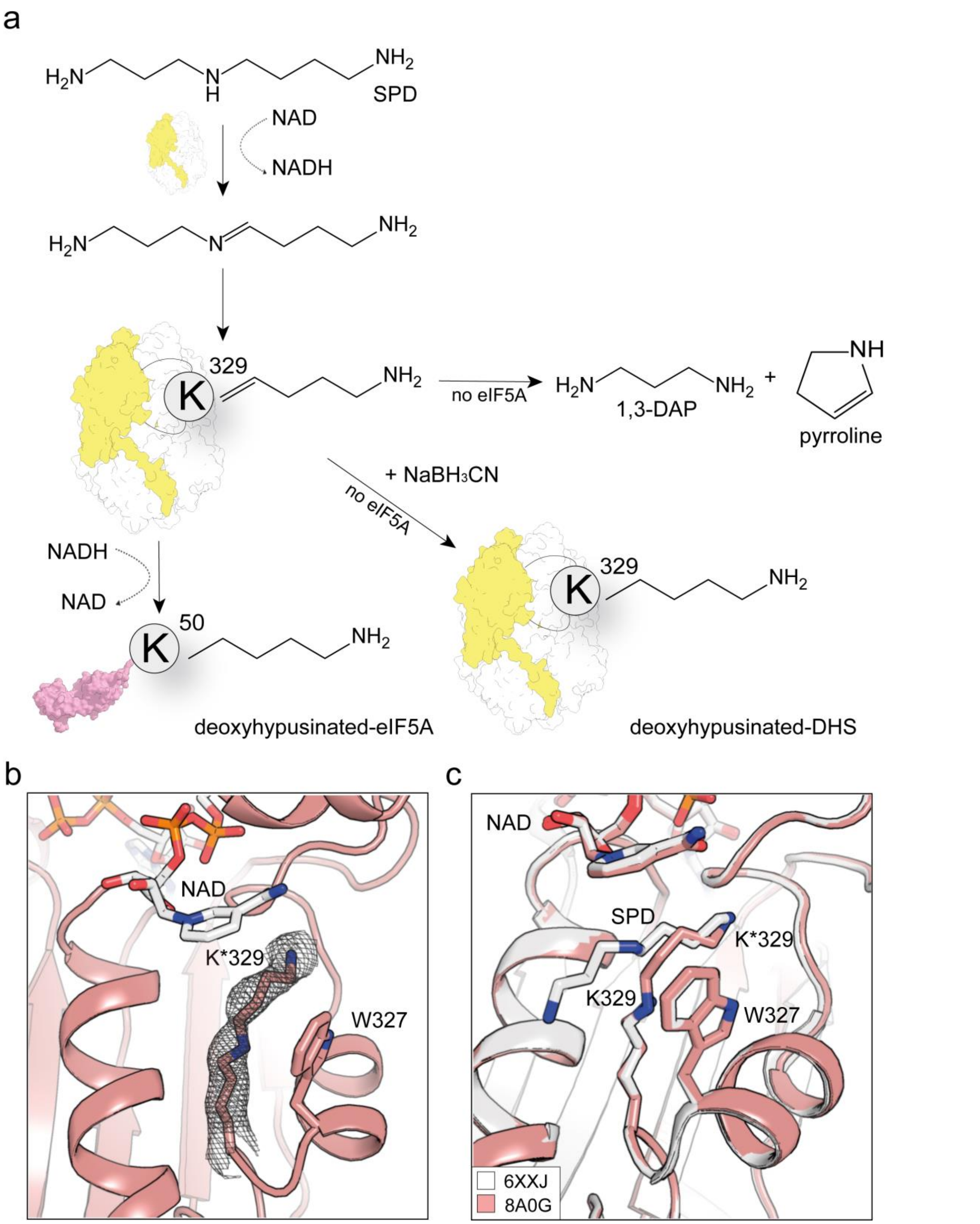

Supplementary Fig. 9. Capturing the reaction intermediate-state analogue. (**a**) Simplified scheme of the DHS-catalyzed reaction. Under normal circumstances (vertical line) the 4-aminobutylo moiety with concomitant reduction of the cofactor is transferred from SPD to K329 in DHS and further to K50 in eIF5A, where upon reduction linked to NADH reoxidation it forms stable deoxyhypusine. A cyclized side product (pyrroline) can emerge as a result of futile redox reaction in the absence of the eIF5A acceptor (mid-horizontal line) or a transition state (4-aminobutyl bound by divalent imine bod to K329) can be trapped by (non-physiological) reduction, rendering a deoxyhypusinated DHS. Such a trapped state differs from the naturally occurring one only in valence of the linkage. (**b**) The electron density map contoured around deoxyhypusinated K329 clearly indicates its elongation. The f2o-fc map is shown as grey mesh around the side chain contoured at 1.0 σ level. (**c**) Superposition of the trapped transition state (pink) on the wild type DHS (white) shows the relative positions of the free SPD and deoxyhypusine. Residue 329, SPD and NAD are shown as sticks, while the main chain of DHS is shown in cartoon representation.

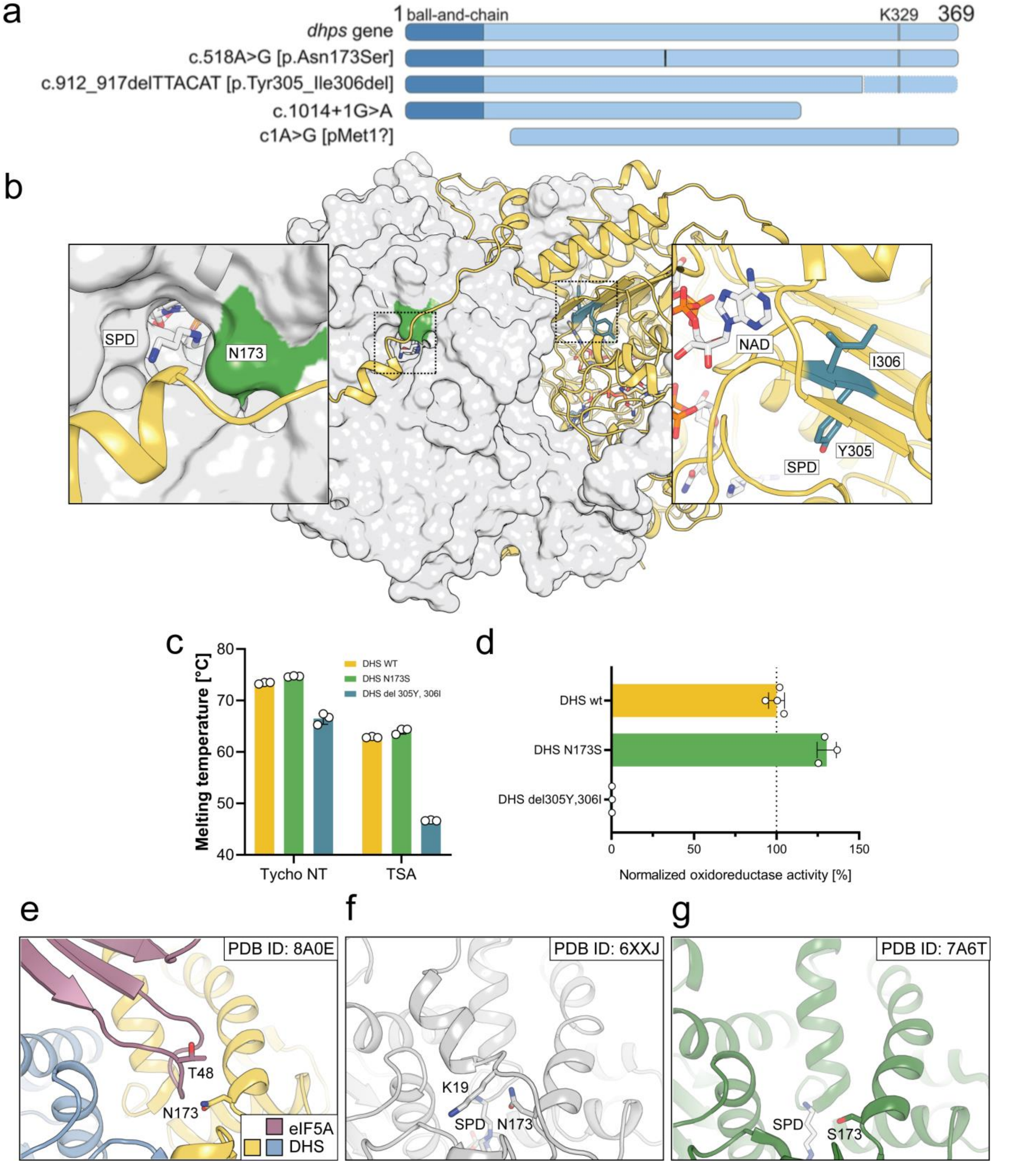

Supplementary Fig. 10. **Clinically-relevant mutations in *dhps* and their impact on the DHS protein.** (**a**) Mutation effects on the DHS encoding gene. (**b**) The overall structure of DHS wt shown as a surface representation with one monomer as a cartoon. The close-ups show the location of pathological mutations on the protein structure. (**c**) Thermal stability of DHS variants assessed by DSF and intrinsic fluorescence measurements with Tycho NT Bars represent the mean ± SD of n=3 independent experiments. Dots represent individual measurements. (d) Oxidoreductase activity of DHS variants relative to DHS wild type. Bars represent the mean ± SD of n=3 independent experiments. Dots represent individual measurements. (**e-g**) Comparison of N173 site between DHS-eIF5A (PDB: 8A0E), DHS wt (6XXJ) and DHS^{N173S} (7AGT). Source data for all panels are provided in Source Data File.

Supplementary Table 1. CryoEM data collection summary.

	**PDB: 8A0E**<br>**EMD-15052**
Data collection and processing	
Voltage (kV)	*300*
Electron exposure (e-/Å²)	40
Defocus range (mm)	-1.0 ÷ -3.5
Pixel size (Å/px)	0.86
Symmetry imposed	C1
Initial images (no.)	12419
Final particles count (no.)	490,582
Map resolution (Å)	2.84
FSC threshold	0.143
Map resolution range (Å)	1.86 ÷ 7.31
Refinement	
Initial model used (PDB code)	6XXJ, 3CPF
Model resolution	2.8
Protein residues (no.)	1526
Ligands (no.)	7
Validation	
RMS deviations	
Bond lengths (Å)	0.013
Bond angles (°)	1.160
MolProbity score	1.04
Clash score	2.52
Ramachandran plot	
Favored (%)	98.27
Allowed (%)	1.73
Outliers (%)	0.00
Model/map fit (volume)	
CC (mask)	0.88
CC (box)	0.73
CC (peaks)	0.71
CC (volume)	0.85
Mean CC for ligands	0.74

Supplementary Table 2. HDX-MS parameters and summary.

	**eIF5A**	**DHS^{K329A}**
**Data collection**		
Protein states	eIF5A	DHS^{K329A}
	eIF5A/DHS^{K329A} complex	eIF5A/DHS^{K329A} complex
Protein concentration	4 µM	
Temperature	25 °C	
Buffer	25 mM HEPES-Na pH 7.5, 150 mM NaCl, 5 mM β-mercaptoethanol	
Incubation time	10, 30, 95, 1,000, 10,000 s	
Replicates	3, technical (same protein batch, separate HDX reactions)	
**Data Analysis**		
Number of peptides	52	197
Sequence coverage	89.3%	98.4%
Redundancy	4.88	6.46
Significance criteria applied	5% relative difference and 0.5 Da absolute difference[2] in HDX	

*The list of identified peptides and their H/D exchange over time is provided in the Source Data file.

Supplementary Table 3. Data collection and refinement statistics. Statistics for the highest-resolution shell are shown in parentheses.

PDB ID	7A6T<br>**DHS N173S in complex with NAD and SPD**	8A0F<br>**DHS K329A in complex with NAD and SPD**	8A0G<br>**DHS with trapped transition state**
Light Source	BESSY 14.1	BESSY 14.1	BESSY 14.3
Wavelength (Å)	0.918	0.918	0.895
Resolution range* (Å)	46.01-1.66 (1.72-1.66)	46.08-1.64 (1.74-1.64)	46.52-1.84 (1.95-1.84)
Space group	$P3_221$	$P3_221$	$P3_221$
Unit cell (Å, °)	105.03 105.03 160.00<br>90 90 120	105.24 105.24 160.22<br>90 90 120	106.95 106.95 161.38<br>90 90 120
Total reflections	1218148 (193939)	1381279 (213024)	922420 (150166)
Unique reflections	120687 (11838)	124222 (18976)	93129 (14866)
Multiplicity	6.3 (16.4)	11.1 (11.2)	9.9 (10.1)
Completeness (%)	99.7 (98.4)	99.1 (94.7)	99.9 (99.5)
Mean I/σ(I)	9.47 (0.55)	11.50 (0.71)	8.58 (0.97)
Wilson B-factor	26.1	33.6	34.8
R-meas (%)	16.2 (361.5)	14.4 (335.5)	20.3 (207.2)
$CC_{1/2}$ (%)	99.8 (46.2)	99.9 (45.3)	99.7 (54.9)
Reflections used in refinement	120518 (11801)	124100 (11268)	92986 (9195)
Reflections used for R-free	2096 (136)	2095 (189)	2097 (206)
R-work (%)	17.1 (44.4)	16.9 (38.3)	15.6 (29.7)
R-free (%)	19.3 (46.8)	18.3 (40.5)	17.1 (32.3)
Number of protein chains in ASU	2	2	2
Number of non-hydrogen atoms	5880	5819	5887
macromolecules	5358	5241	5471
ligands	142	120	111
solvent	380	458	305
Protein residues	676	653	680
RMS(bonds)	0.012	0.012	0.015
RMS(angles)	1.43	1.47	1.66
Ramachandran favoured (%)	98.3	98.6	98.8
Ramachandran allowed (%)	1.7	1.4	1.1
Ramachandran outliers (%)	0.0	0.0	0.1
Rotamer outliers (%)	0.0	0.5	0.7
Clashscore	4.2	2.8	5.1
Average B-factor	38.4	35.1	37.6
macromolecules	37.6	34.3	37.4
ligands	41.6	33.8	32.2
solvent	45.8	44.8	42.8

* Resolution limits according to an I/σ(I) of 2 are: 1.91 Å for 7A6T, 1.84 Å for 8A0F, 1.99 Å for 8A0G.

Supplementary References

1 Weis, D. D. *et al. J. Am. Soc. Mass Spectrom.* 17, 1498–1509 (2006)

2 Houde, D. *et al. J. Pharm. Sci.* 100, 2071–2086 (2011)
