# Supplementary material for: Cryo-EM structure of human eIF5A-DHS complex reveals the molecular basis of hypusination-associated neurodegenerative disorders
Source: Nat Commun. 2023 Mar 27;14:1698. doi: 10.1038/s41467-023-37305-2 (PMC10042821; doi:10.1038/s41467-023-37305-2)
Supplement: Supplementary file 3 — Description of Additional Supplementary Files [file 41467_2023_37305_MOESM3_ESM.pdf]

Description of Additional Supplementary Files

File Name: Supplementary Movie 1
Description: Visualisation of deoxyhypusination reaction.
