# Supplementary material for: Cryo-EM structure of human eIF5A-DHS complex reveals the molecular basis of hypusination-associated neurodegenerative disorders
Source: Nat Commun. 2023 Mar 27;14:1698. doi: 10.1038/s41467-023-37305-2 (PMC10042821; doi:10.1038/s41467-023-37305-2)
Supplement: Supplementary file 5 — Reporting Summary [file 41467_2023_37305_MOESM5_ESM.pdf]

nature portfolio

Corresponding author(s): Przemyslaw Grudnik

Last updated by author(s): Feb 27, 2023

Reporting Summary

Nature Portfolio wishes to improve the reproducibility of the work that we publish. This form provides structure for consistency and transparency in reporting. For further information on Nature Portfolio policies, see our Editorial Policies and the Editorial Policy Checklist.

Statistics

For all statistical analyses, confirm that the following items are present in the figure legend, table legend, main text, or Methods section.

n/a	Confirmed	
☐	☒	The exact sample size (*n*) for each experimental group/condition, given as a discrete number and unit of measurement
☐	☒	A statement on whether measurements were taken from distinct samples or whether the same sample was measured repeatedly
☒	☐	The statistical test(s) used AND whether they are one- or two-sided<br>*Only common tests should be described solely by name; describe more complex techniques in the Methods section.*
☒	☐	A description of all covariates tested
☐	☒	A description of any assumptions or corrections, such as tests of normality and adjustment for multiple comparisons
☐	☒	A full description of the statistical parameters including central tendency (e.g. means) or other basic estimates (e.g. regression coefficient) AND variation (e.g. standard deviation) or associated estimates of uncertainty (e.g. confidence intervals)
☒	☐	For null hypothesis testing, the test statistic (e.g. *F*, *t*, *r*) with confidence intervals, effect sizes, degrees of freedom and *P* value noted<br>*Give P values as exact values whenever suitable.*
☒	☐	For Bayesian analysis, information on the choice of priors and Markov chain Monte Carlo settings
☒	☐	For hierarchical and complex designs, identification of the appropriate level for tests and full reporting of outcomes
☒	☐	Estimates of effect sizes (e.g. Cohen's *d*, Pearson's *r*), indicating how they were calculated

Our web collection on statistics for biologists contains articles on many of the points above.

Software and code

Policy information about availability of computer code

Data collection	MxCube2, EPU v.2.10.0.1941REL (Thermo Fisher), MO.Control v.1.6 (Nanotemper), Unicorn 7.0.0.953 (GE Healthcare), Azure Biosystems v.1.5.0.0518, Tycho NT.6 software v.1.2.0.750, Bio-Rad CFX Manager 3.0, Lab Solutions RF v.1.15, SoftMax Pro Software v.5.4.5.000 & v.7.1, AcquireMP 2.3.0 (Refeyn), G2-Si HDMS mass spectrometer software (Waters)
Data analysis	XDSAPP3 v1.8, WinCOOT 0.8.9.1, PyMOL v.1.7, Phenix1.19.2-4158, MolProbity, PAIRREF, Durchlichtelektronenmikroskopiebilddatenentzerrungswerkzeug v.1.0.9, CryoSPARC v.3.3.1, UCSF ChimeraX 1.1, MO.Affinity Analysis (Nanotemper), Excel 365, GraphPad Prism 8, DiscoverMP 2.3.0 (Refeyn), ProteinLynx Global SERVER (PLGS, Waters), DynamX 3.0 software (Waters)

For manuscripts utilizing custom algorithms or software that are central to the research but not yet described in published literature, software must be made available to editors and reviewers. We strongly encourage code deposition in a community repository (e.g. GitHub). See the Nature Portfolio guidelines for submitting code & software for further information.

Data

Policy information about availability of data

All manuscripts must include a data availability statement. This statement should provide the following information, where applicable:

- Accession codes, unique identifiers, or web links for publicly available datasets
- A description of any restrictions on data availability
- For clinical datasets or third party data, please ensure that the statement adheres to our policy

Data supporting the findings of this study are available within the article and its Supplementary Figures.
The structure coordinates are deposited in the Protein Data Bank with the PDB IDs 8A0E (eIF5A-DHS), 8A0F (DHSK329A), 8A0G (DHS wt with trapped transition state), and 7A6T (DHS N173S in complex with NAD and SPD). The cryo-EM density map for the complex is deposited in EMDB with the ID: EMD-15052. HDX-MS data have been deposited to the ProteomeXchange Consortium via the PRIDE partner repository with the dataset identifier PXD040460. Source data including uncropped Western Blots are provided with this paper.

Additional details on datasets and protocols that support the findings of this study will be made available by the corresponding author upon reasonable request.

Human research participants

Policy information about studies involving human research participants and Sex and Gender in Research.

Reporting on sex and gender	n/a
Population characteristics	n/a
Recruitment	n/a
Ethics oversight	n/a

Note that full information on the approval of the study protocol must also be provided in the manuscript.

Field-specific reporting

Please select the one below that is the best fit for your research. If you are not sure, read the appropriate sections before making your selection.

☒ Life sciences ☐ Behavioural & social sciences ☐ Ecological, evolutionary & environmental sciences

For a reference copy of the document with all sections, see nature.com/documents/nr-reporting-summary-flat.pdf

Life sciences study design

All studies must disclose on these points even when the disclosure is negative.

Sample size	In general, no statistical methods were used to determine sample size. Sample size was determined based on standards for biochemistry and structural biology studies, attempting to have a minimum of N = 3 replicates with sufficient reproducibility. For cryoEM analysis, details of the sample size are listed in Sup. Data Tab.1. For crystallography, the sample size was chosen to ensure completness of the dataset (see Ext. Data Table 3). Additionally, samples from different protein batches were analyzed.
Data exclusions	For cryoEM analysis, standard 2D and 3D classifications were performed.<br>For MST analysis, in several cases outlier points that differs significantly from other observations, were excluded from the analysis.<br>For HDX analysis, peptides which could not be annotated properly were excluded from the analysis.<br>For the rest of experiments, there were no data exclusions.
Replication	For biochemical experiments, at least 3 standard replication experiments were performed with enough reproducibility. Attempts of data replication were, therefore, successful.
Randomization	No randomization was required for the experiments presented in the study as the concept is meaningless for the type of analyses employed.
Blinding	No blinding was required for the experiments presented in the study, as the concept is meaningless for the type of analyses employed.

Reporting for specific materials, systems and methods

We require information from authors about some types of materials, experimental systems and methods used in many studies. Here, indicate whether each material, system or method listed is relevant to your study. If you are not sure if a list item applies to your research, read the appropriate section before selecting a response.

Materials & experimental systems

n/a	Involved in the study
☐	☒ Antibodies
☒	☐ Eukaryotic cell lines
☒	☐ Palaeontology and archaeology
☒	☐ Animals and other organisms
☒	☐ Clinical data
☒	☐ Dual use research of concern

Methods

n/a	Involved in the study
☒	☐ ChIP-seq
☒	☐ Flow cytometry
☒	☐ MRI-based neuroimaging

Antibodies

Antibodies used	primary rabbit FabHpu98 antibody - PABL- 582 (Creative Biolabs);<br>secondary anti-rabbit-IgG horse-radish, peroxidase-conjugated antibody - 7074 (Cell signalling)
Validation	https://doi.org/10.1016/j.jmb.2016.01.006
